# Streptolysin O and its Co-Toxin NAD-glycohydrolase Protect Group A *Streptococcus* from Xenophagic Killing

**DOI:** 10.1371/journal.ppat.1003394

**Published:** 2013-06-06

**Authors:** Maghnus O'Seaghdha, Michael R. Wessels

**Affiliations:** Division of Infectious Diseases, Boston Children's Hospital and Department of Pediatrics, Harvard Medical School, Boston, Massachusetts, United States of America; New York Medical College, United States of America

## Abstract

Group A *Streptococcus* (*Streptococcus pyogenes* or GAS) causes pharyngitis, severe invasive infections, and the post-infectious syndromes of glomerulonephritis and rheumatic fever. GAS can be internalized and killed by epithelial cells *in vitro*, a process that may contribute to local innate defense against pharyngeal infection. Secretion of the pore-forming toxin streptolysin O (SLO) by GAS has been reported to stimulate targeted autophagy (xenophagy) upon internalization of the bacteria by epithelial cells. Whereas this process was associated with killing of GAS in HeLa cells, studies in human keratinocytes found SLO production enhanced intracellular survival. To reconcile these conflicting observations, we now report in-depth investigation of xenophagy in response to GAS infection of human oropharyngeal keratinocytes, the predominant cell type of the pharyngeal epithelium. We found that SLO expression was associated with prolonged intracellular survival; unexpectedly, expression of the co-toxin NADase was required for this effect. Enhanced intracellular survival was lost upon deletion of NADase or inactivation of its enzymatic activity. Shortly after internalization of GAS by keratinocytes, SLO-mediated damage to the bacteria-containing vacuole resulted in exposure to the cytosol, ubiquitination of GAS and/or associated vacuolar membrane remnants, and engulfment of GAS in LC3-positive vacuoles. We also found that production of streptolysin S could mediate targeting of GAS to autophagosomes in the absence of SLO, a process accompanied by galectin 8 binding to damaged GAS-containing endosomes. Maturation of GAS-containing autophagosome-like vacuoles to degradative autolysosomes was prevented by SLO pore-formation and by SLO-mediated translocation of enzymatically active NADase into the keratinocyte cytosol. We conclude that SLO stimulates xenophagy in pharyngeal keratinocytes, but the coordinated action of SLO and NADase prevent maturation of GAS-containing autophagosomes, thereby prolonging GAS intracellular survival. This novel activity of NADase to block autophagic killing of GAS in pharyngeal cells may contribute to pharyngitis treatment failure, relapse, and chronic carriage.

## Author Summary

Group A *Streptococcus* (*Streptococcus pyogenes* or GAS) is the agent of streptococcal pharyngitis (strep throat), invasive infections such as necrotizing fasciitis and streptococcal toxic shock, and post-infectious complications including rheumatic heart disease. Epithelial cells internalize and kill GAS *in vitro* and may contribute to local innate immune defense in the human pharynx. We now find that production of the secreted pore-forming toxin streptolysin O (SLO) triggered targeted autophagy (termed xenophagy) of GAS in human oropharyngeal keratinocytes, but also enhanced GAS intracellular survival. Increased GAS survival was dependent both on pore-formation by SLO and on SLO-mediated translocation of an enzymatically active co-toxin, NAD-glycohydrolase, into the keratinocyte cytosol. The survival-enhancing effect of both toxins was associated with inhibition of lysosomal fusion with GAS-containing autophagosomes to form functional degradative autolysosomes. These findings reveal a novel coordinated role of two streptococcal toxins in protecting GAS from xenophagic killing and enhancing intracellular survival. Prolonged GAS intracellular survival may contribute to pharyngitis treatment failure, relapse, and chronic carriage.

## Introduction

The human-specific pathogen *Streptococcus pyogenes* (Group A *Streptococcus* or GAS) is responsible for common localized infections such as pharyngitis and impetigo as well as less common, but potentially life threatening, conditions including necrotizing fasciitis and streptococcal toxic shock [1]. The pharynx is thought to be the site of initial colonization not only for pharyngitis, but also in most cases of invasive infection. In addition, persistence or recrudescence of pharyngeal colonization has been associated with treatment failures and with relapses after completion of antibiotic therapy for streptococcal pharyngitis [2], [3]. In the 1990s, studies from several laboratories demonstrated that GAS can enter epithelial cells *in vitro* [4], [5], [6], [7], [8], [9]. It has been suggested that survival of the bacteria within pharyngeal or tonsillar epithelial cells *in vivo* may contribute to persistence, as intracellular organisms are protected from many antimicrobial agents and host immune effectors such as complement and antibodies. In support of this view, intracellular GAS have been identified by microscopy and isolated in cultures of surgical specimens of human tonsils, even after treatment of the excised tissue with antibiotics to kill extracellular bacteria [10].

*In vitro* studies suggest that GAS do not proliferate within epithelial cells [4], [9], [11]; however, viable bacteria have been recovered from infected cell lines *in vitro* and from clinical specimens of excised tonsillar tissue days after antibiotic treatment, results consistent with prolonged survival of a subpopulation of GAS in an intracellular niche [9], [10], [12]. Eventual emergence of viable organisms into the extracellular milieu could serve as a source for relapsing or recurrent infection or transmission to others. The concept of pharyngeal epithelial cells as a possible sanctuary site for GAS persistence has stimulated interest in characterizing the cell biology of internalization and intracellular trafficking of GAS.

Studies from our laboratory and by others have implicated the secreted GAS toxin streptolysin O (SLO) as a critical factor modulating the interaction of GAS with epithelial cells *in vitro* [13], [14], [15], [16], [17]. SLO is a member of the cholesterol-dependent cytolysins (CDCs), a large family of related toxins produced by many species of gram-positive bacteria. CDCs bind to cholesterol-containing membranes where they oligomerize and insert into the lipid bilayer to form large pores [18], [19]. Certain CDCs have important biological functions beyond simply cytolytic activity; examples include escape of *Listeria monocytogenes* from the phagosome into the cytosol of macrophages mediated by listeriolysin O and Toll-like receptor signaling by pneumolysin, a product of *S. pneumoniae*, and by other CDCs [20], [21], [22]. Nakagawa *et al*. discovered that SLO production by GAS stimulated association of internalized bacteria with autophagosome-like vacuoles in HeLa cells and promoted killing of the internalized bacteria [16]. In a follow up study, Sakurai *et al*. reported more efficient intracellular killing of wild-type GAS compared to a SLO-negative mutant after internalization into HeLa cells [17]. By contrast, Hakansson *et al*. found significantly greater intracellular survival of SLO-producing GAS in human oropharyngeal keratinocytes *in vitro* compared to an isogenic SLO-negative mutant [14]. The apparently contradictory results of these studies on the effect of SLO on intracellular survival of GAS might reflect differences in GAS strains, differences in epithelial cell lines, and/or differences in experimental conditions used in previous studies.

In this study, we investigated in greater depth the role of SLO in inducing targeted anti-bacterial autophagy (xenophagy) in response to GAS infection of human oropharyngeal keratinocytes, cells representative of the usual site of colonization and infection with GAS. We found that xenophagy was stimulated by SLO, but also by streptolysin S (SLS); however, functional maturation of GAS-containing autophagosomes was inhibited by the pore-forming activity of SLO and by translocation of the co-toxin, NAD-glycohydrolase (NADase). The coordinated actions of SLO and NADase prolonged GAS intracellular survival by preventing effective trafficking of GAS to degradative autolysosomes.

## Results

### Streptolysin O expression is associated with enhanced intracellular survival of GAS in oropharyngeal keratinocytes

Alternative experimental models have yielded conflicting results with respect to the effect of SLO on intracellular survival of GAS after internalization by epithelial cells *in vitro*. To reconcile these earlier observations and to assess more thoroughly the long-term outcome of GAS internalized within pharyngeal epithelial cells, we monitored the intracellular survival of GAS in OKP7 human oropharyngeal keratinocytes over a 24-hour period. Intracellular CFU were assessed by quantitative culture at various time points post-infection and were expressed as a percentage of the intracellular CFU recovered at 2 h. The mean number of intracellular bacteria recovered at 2 h was greater for the SLO-negative mutant strain 188SLO- (3.3×10^4^ CFU/monolayer) than for the parental strain 188 (3.1×10^3^ CFU/monolayer), as reported previously [14], [15].

**Figure 1:**
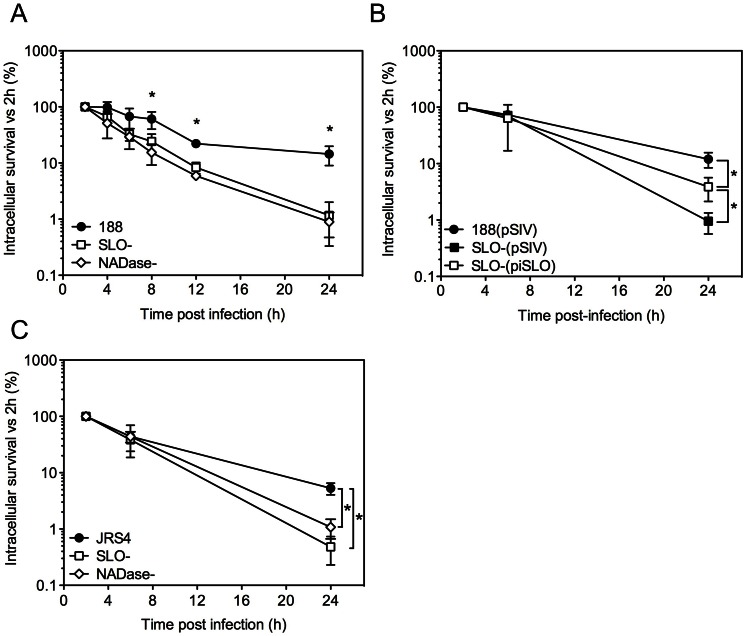
SLO and NADase promote intracellular survival of GAS in oropharyngeal keratinocytes. **A.** Intracellular survival of GAS strains 188, 188SLO- (SLO-), and 188NADase- (NADase-). Intracellular CFU were quantified at 2 h, 4 h, 6 h, 8 h, and 24 h, and intracellular survival was calculated as the percent viable CFU compared to 2 h. **B.** Intracellular survival of GAS strain 188SLO- that was partially complemented from plasmid piSLO (SLO-(piSLO)), strain 188 and 188SLO- that contained the empty complementation vector, 188(pSIV4) and SLO-(pSIV4), respectively. **C.** Intracellular survival of GAS strain JRS4, JRS4SLO- (SLO-), and JRS4NADase- (NADase-). Experiments were performed in triplicate and values represent the mean of three independent experiments ± SD. *, P<0.001.

Comparison of the mean intracellular survival of GAS strain 188 and its isogenic SLO-negative mutant, 188SLO-, revealed 67% survival of parent strain 188 at 6 h, 22% at 12 h, and 14% survival at 24 h. By contrast, intracellular survival of 188SLO- was 33% at 6 h, 8% at 12 h, and only 1% at 24 h (Figure 1A). Thus, production of SLO was associated with a 14-fold advantage in intracellular survival 24 hours after infection. To confirm that the improved survival phenotype required SLO production, we performed similar experiments with strain 188SLO-(piSLO) in which the deletion of *slo* is partially complemented *in trans* by expression of *slo* from a plasmid [15]. SLO hemolytic activity of 188SLO-(piSLO) is approximately 10% of that associated with strain 188 upon IPTG induction of SLO expression [15]. Partial restoration of SLO production in 188SLO-(piSLO) resulted in a 4-fold increase in intracellular survival at 24 h relative to that of 188SLO- containing the vector alone (188SLO-(pSIV)) (Figure 1B). We observed a similar trend in enhanced intracellular survival associated with SLO production for the M type 6 strain JRS4 used in the studies of Nakagawa *et al*. and Sakurai *et al*. [16], [17]: intracellular survival of wild-type JRS4 at 24 h was more than 5-fold higher than that of JRS4SLO- (Figure 1C). We conclude from these results that SLO production enhances GAS survival in human oropharyngeal keratinocytes, a cell type representative of the local environment occupied by GAS during human infection.

In striking contrast, similar experiments in HeLa cells revealed more efficient killing of the SLO-producing parent strain 188 compared to its SLO-negative mutant (Supporting Information, Figure S1A). However, SLO-producing GAS were markedly more cytotoxic to HeLa cells, so it is likely that the low recovery of viable intracellular SLO-producing GAS reflects loss of HeLa cell integrity and exposure of internalized GAS to antibiotic killing (Figure S1B,C). Thus, the effect of SLO on GAS intracellular survival depends on epithelial cell type: SLO production was associated with reduced survival in HeLa cervical carcinoma cells, but increased survival in oropharyngeal keratinocytes.

#### SLO is not essential for GAS-induced autophagy

**Figure 2:**
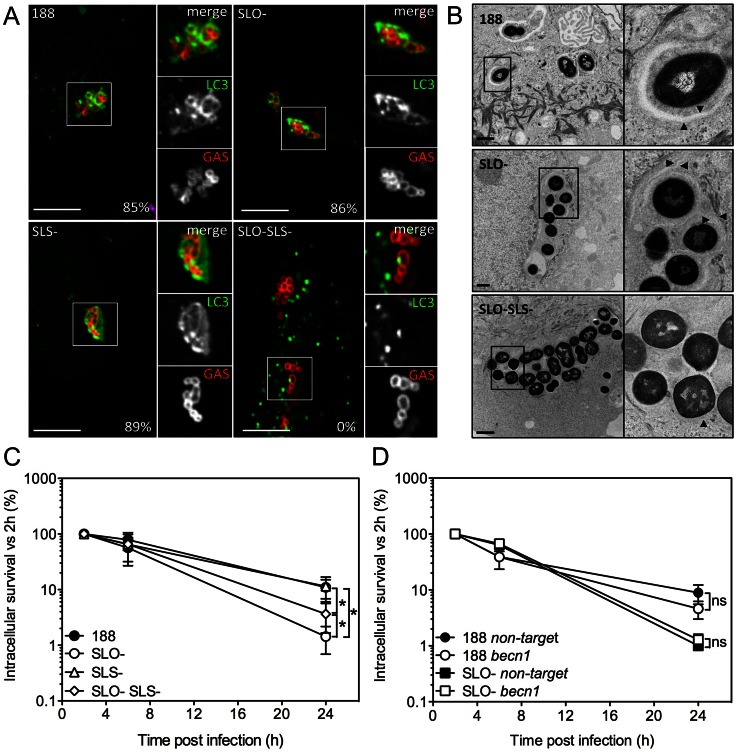
Xenophagy in oropharyngeal keratinocytes can be stimulated by SLO or SLS and is not associated with killing of intracellular GAS. **A.** Confocal microscopy of the association between EGFP-LC3 and GAS strain 188, 188SLO- (SLO-), 188SLS- (SLS-), or 188SLO-SLS- (SLO-SLS-). Immunofluorescent staining distinguished intracellular (Alexa-568, red) from extracellular (Alexa-568 and Alexa-660, red and blue, respectively) GAS. Scale bar = 10 µm. The percent of intracellular GAS that were associated with EGFP-LC3 at 3 h post-infection is shown for each strain. **B.** Electron microscopy of GAS strains 188, 188SLO-, and 188SLO-SLS- at 3 h post-infection. Arrowheads indicate the presence of double or multiple membranes (188 and 188SLO-) or single membranes (188SLO-SLS-) partially or completely surrounding bacteria. Vacuolar compartments associated with 188 and 188SLO- also contain cytosolic material. **C.** Intracellular survival of GAS strains 188, 188SLO-, 188SLS-, and 188SLO-SLS-. *, P<0.05. **D.** Intracellular survival of 188 or 188SLO- in the presence or absence of Beclin1 knockdown.

In HeLa cells, secretion of SLO stimulates the formation of autophagosome-like vacuoles that surround intracellular GAS and appear to mediate bacterial killing, whereas GAS-associated autophagosomes were not observed in cells infected with an SLO-deficient strain [16], [17]. In light of our apparently contradictory finding that SLO production is associated with GAS resistance to intracellular killing in oropharyngeal keratinocytes, we investigated the induction of autophagy/xenophagy by GAS in keratinocytes expressing EGFP-LC3, a marker of the autophagosomal membrane. Unexpectedly, we observed LC3-positive vacuoles surrounding intracellular GAS not only in cells infected with parent strain 188 but also in cells harboring the SLO-deficient strain 188SLO- (Figure 2A). Electron microscopy confirmed these observations: strains 188 and 188SLO- were localized to double or multiple membrane-bound structures that also contained cytosolic material (Figure 2B). That both GAS strains were associated with autophagosome-like compartments indicated that SLO is not required for GAS to induce xenophagy in oropharyngeal keratinocytes. A similar pattern of co-localization was observed for cells infected with GAS strain JRS4 or JRS4SLO-: both strains were found in LC3-positive compartments (Figure S2A). By contrast, in HeLa cells, association of intracellular GAS with EGFP-LC3 was dependent on SLO, as 188SLO- did not associate with autophagosomes, a result in agreement with previous studies (Figure S2B) [16], [17]. Thus, in oropharyngeal keratinocytes, internalization of GAS is associated with induction of xenophagy, whether or not the internalized strain produces SLO.

For strain 188, 40% of intracellular bacteria were associated with LC3 at 1 h post-infection, and by 3 h, 85% were in LC3-positive compartments. Association of 188SLO- with LC3 was initially higher, with 72% of bacteria in autophagosomes at 1 h, and by 3 h, 86% were associated with LC3 (Figure 2A,B).

#### Xenophagy can be induced by SLO or by streptolysin S, and induction of xenophagy does not correlate with the efficiency of intracellular killing of GAS

Induction of xenophagy in response to intracellular bacterial pathogens is commonly associated with damage to the bacterial vacuole membrane by products of the invading organism. Accordingly, we speculated that SLS, another pore-forming toxin produced by GAS, might be responsible for stimulating xenophagy in oropharyngeal cells infected with SLO-deficient GAS. To investigate this hypothesis, we infected oropharyngeal keratinocytes expressing EGFP-LC3 with the double mutant strain 188SLO-SLS- that expresses neither SLO nor SLS, or with 188SLS-, which still produces SLO. 188SLS- associated with LC3-positive compartments to the same extent as 188 (89% of intracellular bacteria at 3 h), but the double mutant 188SLO-SLS- was never found within autophagosome-like compartments by fluorescence microscopy or electron microscopic analysis (Figure 2A,B). Furthermore, analysis of autophagy induction by Western blot revealed that exposure of OKP7 cells to GAS strains expressing SLO (and to a lesser extent, SLS), or to recombinant SLO correlated with a clear increase in the relative abundance of LC3-II, the form of LC3 associated with autophagosomes, whereas relative LC3-II abundance in cells exposed to 188SLO-SLS- was indistinguishable from that in uninfected cells (Figure S2C). These results indicate that production of either SLO or SLS is sufficient to trigger a xenophagic response to GAS in oropharyngeal keratinocytes.

The fact that a GAS strain deficient in both SLO and SLS failed to induce xenophagy provided an opportunity to investigate whether xenophagy is associated with increased intracellular killing of GAS. Intracellular survival rates of 188 and 188SLS- were similar, and survival of both strains was significantly greater than that of 188SLO- or 188SLO-SLS- (Figure 2C). We observed approximately 2-fold greater survival of 188SLO-SLS- compared to 188SLO-. The fact that 188SLO-SLS- fails to induce xenophagy, yet is killed more efficiently than 188 or 188SLS- (that do induce xenophagy) indicates that xenophagy is not a required step in targeting intracellular GAS for efficient killing. We also assessed intracellular survival of GAS in keratinocytes treated with siRNA targeted to *becn1*, which encodes Beclin 1, a component of the class III phosphatidylinositol-3-kinase complex that is required for autophagy (Figure S3) [23], [24]. Knock-down of Beclin 1 had no effect on intracellular survival of 188 or 188SLO- (Figure 2D). Taken together, these data indicate that induction of xenophagy is not required for efficient killing of GAS that are deficient in SLO and SLS, whereas SLO-producing GAS exhibit enhanced intracellular survival despite inducing xenophagy.

#### SLO-mediated translocation of NADase into keratinocytes protects GAS from intracellular killing

**Figure 3:**
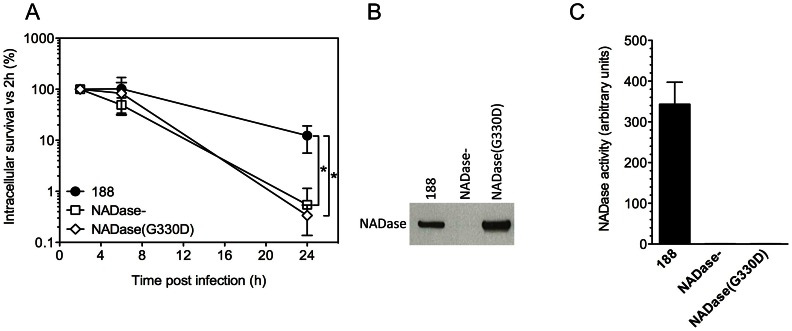
Expression of active NADase plays a central role in the intracellular survival of GAS in oropharyngeal keratinocytes. **A.** Intracellular survival of GAS strains 188, 188NADase-, and 188NADase(G330D). Deletion of the gene encoding NADase or inactivation of NADase by the amino acid substitution G330D resulted in a 20- to 40-fold reduction in intracellular survival compared to parent strain 188. *, P<0.001. NADase western blot (**B**) and activity measurements (C) of culture supernatants from GAS strains 188, 188NADase-, and 188NADase(G330D).

SLO is expressed from an operon that also encodes NADase and its intracellular inhibitor, Immunity Factor for *Streptococcus pyogenes* NADase (IFS) [25], [26]. SLO and NADase are functionally linked in that SLO is required for the translocation of NADase across cholesterol-containing membranes [27], [28]. Since SLO-negative strains of GAS are unable to translocate NADase, the reduced intracellular survival of such strains could be due to the absence of SLO or to failure to translocate NADase into keratinocytes, or both. To specifically address the potential role of NADase, we tested intracellular survival of strain 188NADase-, an isogenic mutant that produces SLO, but not NADase [27]. Deletion of NADase was associated with an increase in bacterial uptake: the mean number of intracellular GAS was 8.7×10^3^ CFU/monolayer at 2 h for strain 188NADase- compared to 3.1×10^3^ CFU/monolayer for strain 188, in agreement with previously published observations [14], [27]. Intracellular survival of the NADase-negative strain was reduced by approximately 20-fold compared to parent strain 188 (Figures 1A and 3A). NADase expression also enhanced intracellular survival of GAS strain JRS4 (Figure 1C).

Translocation of NADase into keratinocytes from extracellular GAS has been shown to result in depletion of cellular NAD^+^ and ATP [29]. To test whether NAD-glycohydrolase activity of the translocated protein is responsible for its protective effect on GAS intracellular survival, we generated mutant strain 188NADase(G330D), a derivative of 188 in which the chromosomal *nga* locus harbors a point mutation coding for amino acid substitution G330D in NADase. This mutation has been associated with enzymatically inactive NADase in clinical isolates of GAS and in a deliberately constructed mutant [30], [31]. Measurement of the NAD-glycohydrolase activity of culture supernatants and western blotting using an anti-NADase antibody confirmed that 188NADase(G330D) secreted similar amounts of NADase protein as parent strain 188, but that the protein was enzymatically inactive (Figure 3B,C). Comparison of the intracellular survival of 188 and 188NADase(G330D) in keratinocytes revealed similarly poor survival of 188NADase(G330D) as 188NADase-, indicating that NAD-glycohydrolase activity is required for NADase to promote GAS intracellular survival (Figure 3A).

As an alternative approach to investigating the role of NADase in enhancing GAS intracellular survival, we used the NADase inhibitor IFS to block the enzymatic activity of NADase produced by parent strain 188. Ordinarily, IFS is produced within the bacterial cell where it acts as an intracellular antitoxin, binding NADase and inhibiting its enzymatic activity until NADase is secreted [25], [26]. We reasoned that IFS also could be used as an inhibitor of enzymatic activity after NADase had been secreted from the bacterial cell. For this purpose, we expressed FLAG-tagged IFS preceded by an N-terminal signal sequence from the streptococcal expression vector pDL278 in GAS strain 188. This strain, 188(pSEC-IFS), secretes IFS constitutively, the result of which is the complete inhibition of NADase activity in culture supernatants despite the production of NADase protein in amounts similar to strain 188 (Figure S4). We tested 188(pSEC-IFS) in intracellular survival assays together with 188 and 188NADase- that contained empty expression vector, pDL278. The intracellular survival of 188(pDL278) and 188NADase-(pDL278) was as expected: deletion of NADase resulted in a 30-fold decrease in intracellular survival. The intracellular survival of 188(pSEC-IFS) was also significantly lower (15-fold) than that of 188, supporting the hypothesis that inactivation of secreted NADase promoted the killing of intracellular GAS (Figure S4).

#### Pore-formation by SLO promotes cytosolic exposure and survival of intracellular GAS

**Figure 4:**
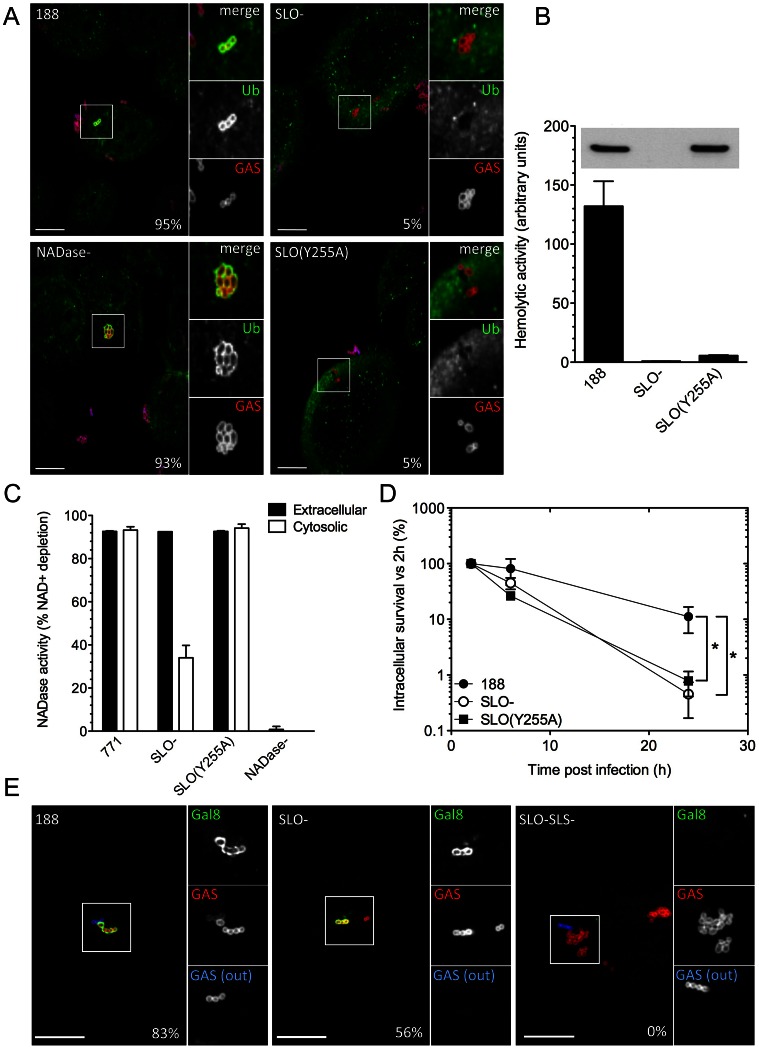
SLO, SLS, and NADase affect xenophagy and GAS intracellular survival. **A.** Confocal microscopy demonstrating the association between ubiquitin (green) and intracellular GAS (red) at 30 min post-infection in keratinocytes infected with GAS strain 188, 188SLO-, 188NADase-, or 188SLO(Y255A). Extracellular GAS were stained blue and red for identification as described in Figure 2. Scale bar = 10 µm. The percentage of intracellular GAS that were associated with ubiquitin is indicated for each strain, based on quantification of at least 100 bacteria in three independent experiments. **B.** Hemolytic activity of culture supernatants of GAS strain 188, 188SLO-, and 188SLO(Y255A). The amino acid substitution Y255A in SLO abrogates the ability of SLO to porate the host cell membrane, demonstrated by the failure of culture supernatants from 188SLO(Y255A) to lyse erythrocytes despite producing wild-type amounts of the variant SLO protein. Inset, corresponding Western blot for SLO in culture supernatants from these strains. **C.** NADase activity in cell culture supernatants and in the cytosol of keratinocytes infected with GAS strain 771, 771SLO-, 771SLO(Y255A), or 771NADase-. Intracellular NADase activity due to translocation by extracellular GAS was identical between 771 and 771SLO(Y255A), but reduced in 771SLO-. **D.** The intracellular survival of 188SLO(Y255A) was significantly lower than that of its parental strain, 188, and similar to that of 188SLO-. *, P<0.01. **E.** Confocal microscopy demonstrating the association between galectin 8 (green) and intracellular GAS (red) in keratinocytes infected for 30 min with GAS strain 188, 188SLO-, or 188 SLO-SLS-. Extracellular GAS (GAS(out)) were stained blue and red for identification as described in Figure 2. Scale bar = 10 µm. The percentage of intracellular GAS that were associated with galectin 8 is indicated for each strain, based on quantification of at least 100 bacteria in three independent experiments. 188SLO-SLS- was never associated with galectin 8 (0%).

The finding that NADase plays a major role in GAS intracellular survival raised the possibility that the importance of SLO in GAS survival might be limited to its ability to translocate NADase into the host cell. In order to test whether the pore-forming activity of SLO also contributes to GAS intracellular survival independently of NADase translocation, we took advantage of the observation of Magassa *et al*. that the pore-forming and NADase-translocating activities of SLO are separable: these authors described an amino acid substitution in SLO, Y255A, that prevented pore-formation and cytolytic activity, but preserved NADase translocation [32]. We introduced this substitution into the chromosomal *slo* locus in strain 188 to generate 188SLO(Y255A). Measurement of the hemolytic titer and SLO production from the culture supernatant of 188SLO(Y255A) confirmed that the mutant strain had lost the capacity to lyse erythrocytes but still produced a similar amount of SLO protein as the parent strain (Figure 4B).

Next, we investigated NADase translocation by the SLO(Y255A) variant. NADase translocation can be measured by determining the NADase activity in the cytosol of cells exposed to extracellular GAS. To do this, we introduced the SLO(Y255A) substitution into strain 950771 (referred to hereafter as 771), the encapsulated wild-type strain from which strain 188 was derived by inactivation of the hyaluronic acid capsule biosynthetic locus [33]. Capsule greatly reduces internalization of GAS, and well-encapsulated strains such as 771 allow accurate measurement of NADase translocation from outside the cell, rather than production of NADase by intracellular GAS [9], [27]. Keratinocytes were incubated for 1.5 h with 771, 771SLO-, 771SLO(Y255A), or 771NADase- in the presence of cytochalasin D to further reduce bacterial internalization, and NADase activity in the extracellular medium and the keratinocyte cytosol was measured. NADase production and secretion by 771, 771SLO-, and 771SLO(Y255A) was similar, as demonstrated by the NADase activities from culture supernatants (Figure 4C). Strains 771 and 771SLO(Y255A) translocated similar amounts of NADase activity into the cytosol of GAS-exposed keratinocytes, whereas intracellular NADase activity due to 771SLO- was greatly reduced and none was detected in the cells exposed to 771NADase- (Figure 4C). These data demonstrate that 771SLO(Y255A) and, by inference, 188SLO(Y255A) are defective in SLO pore-formation but not in NADase translocation.

In considering the possible importance of SLO pore-formation in the intracellular survival of GAS, we first investigated whether SLO produced by intracellular GAS damaged the membrane of the bacteria-containing vacuole. Nakagawa *et al*. proposed that shortly after invasion of epithelial cells, GAS escape early endosomes and enter the cytosol in a SLO-dependent manner [16]. To test directly whether internalized GAS disrupt the integrity of the endosomal membrane, we investigated whether GAS become ubiquitinated after internalization. Consistent with the model of SLO-mediated escape into the host cytosol, we found that most intracellular organisms of strain 188 co-localized with ubiquitin within 30 min of exposure to keratinocytes (>95%). On the other hand, fewer than 5% of intracellular 188SLO- bacteria were associated with ubiquitin (Figure 4A). We speculated that the ubiquitination of 188SLO-, albeit at a much-reduced level compared to 188, might be explained by vacuolar membrane damage and consequent cytosolic exposure due to the production of SLS. Indeed, intracellular 188SLO-SLS- bacteria were never observed to be associated with ubiquitin (not shown). Strain 188SLS-, which produces wild-type amounts of SLO, behaved similarly to the parental strain with respect to ubiquitin-binding (90% at 30 min). As with parent strain 188, intracellular 188NADase- bacteria also became ubiquitinated, a result that implies that NADase is not required for the vacuolar membrane damage that exposes internalized GAS to the host cell cytosol. The pattern of ubiquitination of intracellular organisms of the SLO-non-pore-forming, but NADase translocation-competent 188SLO(Y255A) was similar to 188SLO-, i.e., only occasional bacteria were associated with ubiquitin (Figure 4A). This observation indicated that pore-formation is critical to the ability of SLO to mediate exposure of internalized GAS to keratinocyte cytosol.

To address the specific involvement of pore formation by SLO in the intracellular survival of GAS, we next compared the intracellular survival of the SLO-non-pore-forming mutant 188SLO(Y255A) with that of parent strain 188 and 188SLO-. The intracellular survival of 188SLO(Y255A) was reduced to a similar extent as 188SLO-, and was reduced approximately 14-fold compared to 188 (Figure 4D), results that support a specific role of SLO-mediated pore formation in evasion of GAS killing by oropharyngeal keratinocytes.

#### SLS mediates xenophagic recognition of GAS via galectin 8, in a ubiquitin-independent manner

In light of our finding that wild-type GAS are exposed to the cytosol through SLO poration of endosomes, it is likely that these bacteria are targeted to autophagy through the well-characterized ubiquitin-NDP52/p62 pathway (for reviews, see [34], [35]). We next sought to understand how, in the absence of SLO-mediated disruption of endosome integrity (and subsequent ubiquitination of liberated GAS), SLS could trigger a xenophagic response to GAS. SLS, like SLO, exhibits hemolytic activity *in vitro*, but does not produce large pores that can be visualized by electron microscopy [36]. It has recently been shown that the cytosolic exposure of glycans normally localized to the interior face of vesicular membranes can result in binding of the cytosolic lectin, galectin 8. By recruiting NDP52, bound galectin 8 targets damaged vesicles for intracellular trafficking to autophagosomes in a ubiquitin-independent manner [37]. This alternative pathway was shown to mediate an autophagic response to membrane-damaging intracellular pathogens such as *Salmonella*, *Listeria*, and *Shigella*. We therefore investigated whether SLS-producing GAS stimulated accumulation of galectin 8, even in the absence of SLO production. Both 188 and 188SLO- associated with galectin 8 within 30 min of uptake by OKP7 cells (83% and 56% of intracellular bacteria, respectively), whereas 188SLO-SLS- was never found to be associated with galectin 8 (Figure 4E). These data support a model in which SLO-deficient GAS damage the integrity of the endosomal vacuole through the membrane-damaging effect of SLS and thus induce the autophagic response in the absence of ubiquitination (Figure 2A). SLO-producing GAS cause sufficient damage to fully expose the bacteria to the cytosol and recruit the ubiquitin pathway. The SLO-SLS- mutant cannot damage endosomes, since it lacks both pore-forming toxins, and does not result in galectin 8 recognition, ubiquitin binding, or trafficking of the bacteria to autophagosomes.

#### SLO and NADase inhibit maturation of GAS-containing autophagosomes

**Figure 5:**
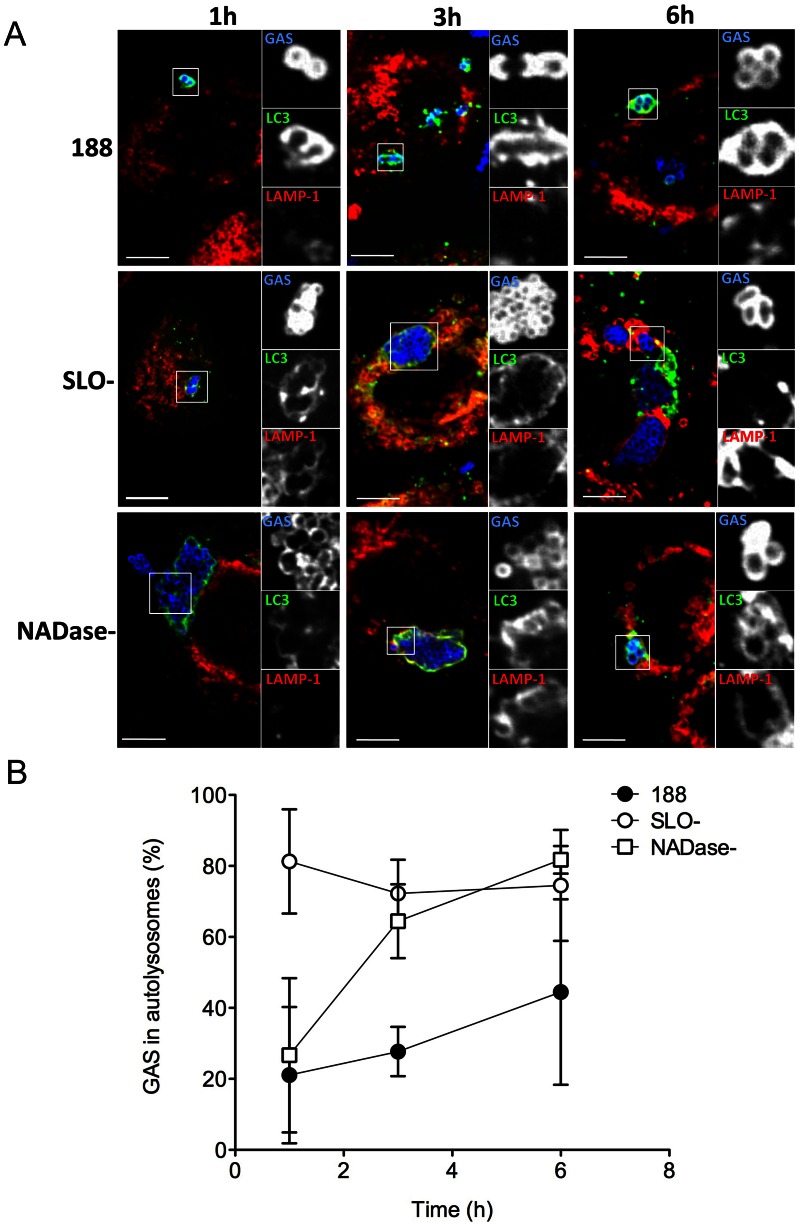
SLO and NADase inhibit lysosomal fusion to GAS-containing autophagosomes in oropharyngeal keratinocytes. **A.** Confocal microscopy of keratinocytes infected with GAS strain 188, 188SLO-, or 188NADase- demonstrating the association of the lysosomal marker LAMP-1 (red) with GAS (blue) contained within EGFP-LC3 (green)-positive compartments at 1 h, 3 h, and 6 h post-infection. Scale bar = 10 µm. **B.** Quantification of the percent of GAS within EGFP-LC3-positive compartments that are co-localized with LAMP-1 at 1 h, 3 h, and 6 h. Data represent mean values from three independent experiments in which at least 100 intracellular GAS were quantified for each time point in each experiment.

The data presented above suggest that optimal GAS survival within human oropharyngeal cells depends on production of both SLO and its co-toxin, NADase. However, these results did not address the mechanism by which toxin production inhibits intracellular killing. A previous study from our group showed that SLO-deficient GAS rapidly co-localized with lysosomal markers after internalization by keratinocytes, whereas SLO-producing GAS did not [14]. Since results of the current investigation showed that both parent strain 188 and SLO- and NADase-deficient GAS are contained within autophagosome-like vacuoles, we reasoned that the differential survival of these strains might reflect the effects of SLO and NADase on the functional maturation of GAS-containing autophagosomes rather than simply induction of xenophagy *per se*. Maturation of autophagosomes into degradative autolysosomes occurs by fusion with lysosomes, which can be detected *in vitro* by co-localization of LC3 with the lysosomal marker LAMP-1. We therefore investigated the temporal association of LAMP-1 with LC3-positive vacuoles containing GAS strains 188, 188SLO-, or 188NADase-. All three GAS strains associated with LC3 shortly after internalization by the host cell (Figure 5A). However, there was a marked difference in the rate of lysosomal fusion: in the case of parent strain 188, 21% of LC3-associated GAS were co-localized with LAMP-1 after 1 h. At 3 h, 28% were co-localized, and by 6 h post-infection, 44% of autophagosomes containing 188 had fused with lysosomes (Figure 5A,B). In contrast, 81% of LC3-associated 188SLO- GAS co-localized with LAMP-1 at 1 h post infection, 72% at 3 h post-infection, and 75% at 6 h (Figure 5A,B). LAMP-1 was also more rapidly associated with autophagosomes harboring 188NADase- compared to parent strain 188, although less so than the SLO-mutant: 27% of autophagosomes containing 188NADase- were positive for LAMP-1 at 1 h post-infection, 64% at 3 h, and 82% at 6 h (Figure 5A,B). We observed a similar pattern for the intracellular trafficking of GAS strains JRS4, JRS4SLO-, and JRS4NADase-. That is, JRS4SLO- and JRS4NADase- rapidly associated with both LC3 and LAMP-1, whereas the co-localization of LC3-positive compartments containing parent strain JRS4 was delayed (not shown). Taken together, these data indicate that the co-expression of SLO and NADase by GAS inhibits maturation of GAS-containing autophagosomes into degradative autolysosomes, and that this delay is associated with prolonged bacterial survival in infected keratinocytes.

## Discussion

The epithelial surface of the human oropharynx represents the primary environmental niche for GAS. The bacteria are adapted to survive in this anatomic site, and, in general, the human host is able to contain GAS infection as localized pharyngitis or asymptomatic carriage. In part, GAS proliferation and spread may be controlled by ingestion and killing of the organisms by oropharyngeal epithelial cells: *in vitro* assays have demonstrated uptake of GAS by oropharyngeal keratinocytes [9], [15], [38]. These studies have concluded that internalized GAS fail to proliferate within keratinocytes and the number of viable intracellular bacteria declines over time. However, some authors have emphasized that a fraction of internalized GAS may survive intracellularly for days, potentially representing a source for relapsing or recurrent infection or persistent carriage despite antibiotic treatment [10], [12].

Nakagawa *et al*. proposed that GAS are not killed by endosomal-lysosomal fusion after entry into epithelial cells, but rather that internalized bacteria escape from the endocytic vacuole into the cytosol where they are captured and degraded by autophagy in a process that depends on SLO [16]. They found that SLO-deficient GAS failed to escape from early endosomes and survived better within HeLa cells than did the SLO-producing parent strain [16], [17]. By contrast, our group reported that SLO production was associated with enhanced GAS survival in oropharyngeal keratinocytes, while SLO-deficient organisms were efficiently killed in a cellular compartment with features of lysosomes including localized immunostaining for LAMP-1 and marked vacuolar acidification [14].

In the present investigation, we examined in greater detail the effects of SLO on intracellular trafficking and long-term survival of GAS in oropharyngeal keratinocytes. We confirmed that production of SLO was associated with a striking increase in intracellular survival 24 hours after initial infection. This effect was not strain-specific as a similar pattern was observed in two independent GAS strains in comparison with their respective SLO-deficient mutants. We also confirmed that internalized GAS were associated with LC3, consistent with their containment within autophagosomes. Electron microscopy revealed that GAS resided in double membrane-bound structures that also contained cytosolic material, consistent with GAS-containing autophagosome-like structures observed in other epithelial cells [16]. Unexpectedly, SLO-deficient strains also co-localized with LC3, whereas a double mutant deficient in both SLO and SLS did not. We found that in the absence of SLO, SLS production caused sufficient endosomal membrane damage for recognition by galectin 8 and subsequent ubiquitin-independent targeting to autophagosomes. Thus, at least two pathways exist for GAS to autophagy, and either cytolytic toxin can damage the endocytic vacuole sufficiently to induce a xenophagic response that targets the hemolysin-producing bacteria. These patterns of cellular response are similar to those observed in cells infected with other bacteria that damage the endocytic or phagocytic vacuole by production of a pore-forming toxin, e.g., listeriolysin O of *Listeria monocytogenes* or the type three secretion system of *Salmonella* Typhimurium [37], [39], [40], [41].

For GAS, induction of targeted autophagy *per se* was not correlated with effective killing of intracellular bacteria: SLO-positive GAS induced xenophagy and survived well, whereas SLO-negative (but SLS-positive) GAS also induced xenophagy, but survived poorly. Furthermore, induction of xenophagy was not required for efficient killing of SLO-negative GAS: a mutant deficient in both SLO and SLS failed to induce xenophagy, but was efficiently killed. Rather, production of SLO and the associated co-toxin NADase appeared to be the critical events in prolonged intracellular survival. The translocation of NADase into keratinocytes has been shown previously to result in profound depletion of intracellular NAD^+^ and ATP and to enhance SLO-mediated cytotoxicity [27], [29]. In the present study, we found that SLO was required for prolonged GAS intracellular survival, both for its ability to translocate NADase into the keratinocyte cytosol and for its vacuolar membrane-damaging activity. NADase was equally required for prolonged GAS survival, as mutants lacking NADase or expressing an enzymatically inactive protein survived poorly despite the membrane-damaging effects of SLO. Further evidence for a key role for NADase came from experiments in which the endogenous inhibitor of NADase was used to inhibit the activity of the enzyme during intracellular infection. Inhibiting NADase activity had a similar effect as inactivating the *nga* gene: it markedly reduced GAS intracellular survival. By depleting cellular energy stores, NADase may inhibit cellular repair of SLO damage to the GAS-containing vacuole. Thus, the expression of both SLO and NADase by wild-type GAS results in delayed and incomplete fusion of LC3-positive GAS-containing vacuoles with lysosomes, a pattern consistent with the observed prolonged survival of strains that produce both toxins.

**Figure 6:**
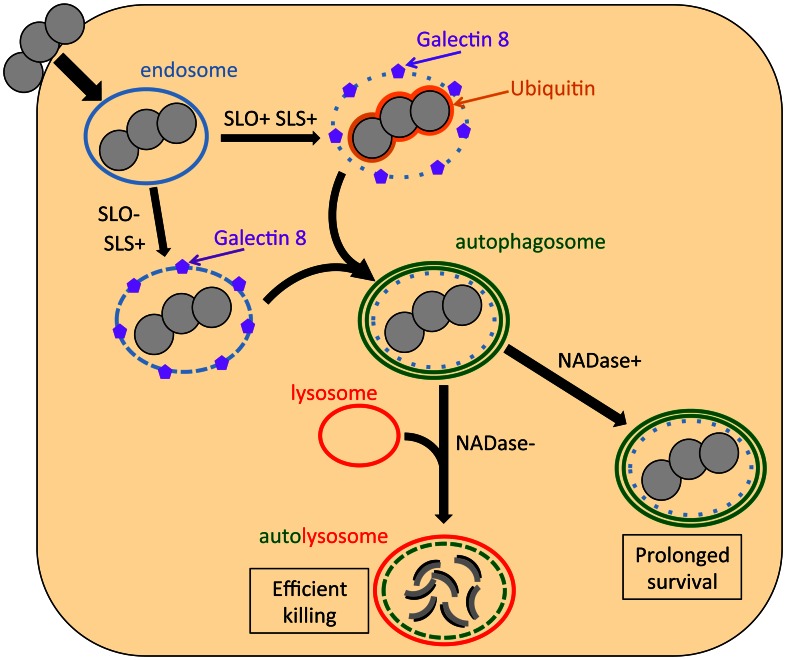
Model of the intracellular fate of GAS in oropharyngeal keratinocytes. Shortly after internalization, GAS are associated with early endosomes. Within minutes, however, damage to the endosomal membrane by the action of SLO and SLS exposes glycans that bind the cytosolic lectin, galectin 8. In addition, SLO produces sufficient damage to the GAS-containing endosome that the bacteria become ubiquitinated upon exposure to the cytosol. Ubiquitin and/or galectin 8 target cytosolic bacteria and damaged endosomes for incorporation into autophagosome-like compartments, which eventually fuse with lysosomes. Lysosomal fusion is delayed or inhibited by SLO pore-formation and SLO-mediated delivery of NADase into the cell cytosol, thus prolonging GAS intracellular survival.

Our results suggest a model for GAS interaction with oropharyngeal epithelial cells in which SLO and NADase are central determinants of the fate of internalized bacteria (Figure 6). Production of the pore-forming toxin SLO (and SLS) damages the membrane of the bacterial vacuole, exposing GAS to the cytosol, which triggers ubiquitin binding to the bacteria or to associated damaged vacuolar membrane. Concurrently, damaged endosomal membrane is recognized by cytosolic galectin 8. The process of endosome disruption by either hemolysin stimulates formation of an autophagosome-like compartment around the bacteria and/or the damaged endosome. Since SLO and NADase are expressed from the same operon, GAS that escape from endosomes via SLO production presumably can secrete NADase directly into the cytosolic milieu prior to capture by the host autophagic machinery. Furthermore, SLO secreted by extracellular bacteria bound to the epithelial cell surface mediates translocation of NADase across the cell membrane into the keratinocyte cytosol. It remains unclear whether NADase is translocated across endosomal or autophagosomal membranes by SLO or by passive diffusion through damaged compartment membranes, or if sufficient amounts of enzyme are delivered to the cytosol by SLO-mediated translocation from extracellular GAS. In either case, once in the host cytosol, the enzymatic activity of NADase inhibits or delays lysosomal fusion with GAS-containing autophagosomes, an effect that promotes prolonged intracellular survival.

The fact that SLO-deficient GAS induced xenophagy but were efficiently killed suggests that vacuolar membrane damage by SLS is sufficient to induce xenophagy in the absence of SLO, but insufficient to prevent rapid lysosomal fusion with the GAS-containing vacuole. Ubiquitination of SLO-deficient GAS was also drastically reduced compared to that of SLO-producing strains, an observation that implies that SLS-mediated membrane injury by itself is insufficient to allow bacterial access to the cytosol. These observations suggest that SLO, through its potent endosomal membrane-damaging activity and/or by translocating NADase, plays a central role in intracellular survival that cannot be replaced by SLS alone. Finally, blocking autophagy by knockdown of Beclin 1 failed to improve intracellular survival of SLO-deficient GAS, so it appears that endosomes containing SLO-deficient GAS are able to rapidly fuse with lysosomes for efficient bacterial killing even if the autophagy pathway is blocked. In conclusion, xenophagy is unnecessary for efficient killing of SLO-deficient GAS and ineffective at killing wild-type GAS.

These findings reveal new insights into the complex molecular dialog between GAS and the human host. Ingestion and killing by pharyngeal epithelial cells appear to contribute to innate defense against GAS infection. For organisms such as GAS that escape from the canonical endocytic pathway, xenophagy represents an alternative strategy of host cells to recapture these pathogens. Although the SLO-mediated damage to the endosomal membrane stimulates a xenophagic response, SLO also prevents functional maturation of GAS-containing autophagosomes by direct injury to the bacterial vacuole and by translocating NADase to the cytosol where it inhibits lysosomal fusion. Together, these observations uncover a novel mechanism for inhibition of xenophagic killing of GAS and suggest that enhancement of intracellular survival through the coordinated action of SLO and NADase contributes to GAS persistence in the human pharynx.

## Materials and Methods

### Reagents

Unless otherwise indicated, reagents were purchased from Sigma. Restriction endonucleases were from Thermo Scientific and cell culture reagents were purchased from Life Technologies. Antibodies to GAS group A carbohydrate, SLO, and NADase have been described previously [29], [42]. Mouse anti-LAMP-1 (CD107a) was from BD Biosciences (555798); mouse anti-ubiquitin (clone FK2) was from Enzo Life Sciences; anti-galectin 8 affinity-purified goat polyclonal IgG (AF1305) was from R&D Systems; rabbit anti-Beclin 1 antibody (NB500-249) was from Novus Biologicals. Unconjugated and fluorescent secondary antibodies were from Santa Cruz and Molecular Probes, respectively. Fluorescent dyes were purchased from Molecular Probes. siRNA reagents were from Dharmacon (Lafayette, CO).

### Bacterial strains and culture conditions

GAS strains used in this study are listed in Table S1. GAS strain 188 is an isogenic unencapsulated mutant of the M type 3 necrotizing fasciitis isolate 950771 [33]. Use of an unencapsulated mutant results in efficient internalization of GAS by human cells *in vitro* because internalization is inhibited by the hyaluronic acid capsule [9]. GAS strains JRS4, JRS4SLO-, and JRS4NADase- were provided by M. Caparon [28], [43], [44]. *Escherichia coli* DH5α was used as a host for molecular cloning (Zymo Research) and was grown in Luria-Bertani (LB) medium (Novagen). GAS were routinely grown in L3 medium with two modifications [45]: the final CaCl_2_ concentration was 0.015% and type 1-S bovine hyaluronidase was omitted. When appropriate, antibiotics were used at the following concentrations: ampicillin at 100 µg/ml, penicillin at 20 µg/ml or 1 µg/ml, spectinomycin at 50 µg/ml, gentamicin at 200 µg/ml, and erythromycin at 200 µg/ml or 1 µg/ml for *E. coli* or GAS, respectively.

### DNA manipulations

Oligonucleotide primers are listed in Table S2. To generate the substitutions Y255A in SLO and G330D in NADase, the codons encoding the relevant residues were mutated by overlap extension PCR. Briefly, using GAS 188 genomic DNA as a template, overlapping oligonucleotides containing the desired mutation (sloY255AF and sloY255AR or ngaG330DF and ngaG330DR) were used in separate PCR reactions with an upstream and downstream primer (slo_up and slo_down or nga_up and nga_down) to generate two PCR products that overlap in the region containing the mutation. This was followed by a second PCR amplification using the external ‘up’ and ‘down’ oligonucleotide primers, using the product of the first PCR as a template, to generate a single recombinant DNA fragment of 1.5 kb (SLO(Y255A)) or 1.3 kb (NADase(G330D)). The external oligonucleotides also contained restriction endonuclease sites (*Bam*HI and *Sal*I) that facilitated directional cloning into the streptococcal shuttle vector, pJRS233 [46]. DNA inserts were verified by DNA sequencing (Genewiz). The recombinant shuttle plasmids were then introduced into GAS strain 188 or 771 by electroporation, and mutant alleles were exchanged for the native chromosomal loci by allelic exchange as described previously [33]. Strain 188SLO-SLS- was constructed by allelic exchange of an internally-deleted *sagA* sequence in strain 188SLO- as described previously [47]. Mutant strains were verified by DNA sequencing of the relevant locus (Genewiz).

To generate pSEC-IFS, two recombinant DNA fragments were cloned in the streptococcal plasmid pDL278 [48] into which the constitutive *guaB* promoter had already been inserted (pDL278-PguaB, a gift from Ioannis Gryllos). The first fragment was amplified from a genomic DNA preparation of strain 188 using the primer pairs SigF and SigR (see Table S1) and comprised the region encoding the signal sequence of SLO. This fragment contained a 3′ *Bam*HI site to facilitate ligation to a 5′ *Bam*HI site within the second PCR fragment, which encoded a FLAG tag linked to *ifs*, generated by PCR using 188 genomic DNA as template and the primers ifsF and ifsR (Table S1).

To generate pBabe/EGFP-LC3, the *Age*I-*Sal*I fragment from pEGFP-LC3 (Addgene plasmid 11546) was ligated into pBabe-puro (Addgene plasmid 1764) that had been digested with *Ngo*MIV and *Sal*I, which produce compatible ends.

RNAi was performed according to the manufacturer's instructions (Dharmacon), and knock-down of Beclin 1 was confirmed by western blot.

### Cell culture

OKP7/bmi1/TERT (OKP7) immortalized primary human soft-palate keratinocytes were a gift of James Rheinwald and were provided through the Harvard Skin Disease Research Center [49]. Keratinocytes were cultured in serum-free keratinocyte medium (KSFM, Life Technologies) supplemented with 50 µg ml^−1^ bovine pituitary extract, 0.1 ng ml^−1^ epidermal growth factor and 0.3 mM calcium chloride. Other cell lines were grown in DMEM containing 10% fetal bovine serum. HeLa cells (ATCC) were transfected with plasmid EGFP-LC3 encoding a fusion protein of human LC3 with EGFP. Cells were transfected by incubation with pEGFP-LC3 and Lipofectamine 2000 (Life Technologies) for 6 hours in Opti-MEM (Life Technologies). Transient expression of EGFP-LC3 in OKP7 cells was achieved by retroviral transduction with viral particles produced by the transfection of HEK293T cells with pBabe/EGFP-LC3 and a three plasmid retroviral system as previously described [50].

### Intracellular survival assay

OKP7 cells were infected at a multiplicity of infection (MOI) of 10 with GAS that had been grown to mid-exponential phase and washed twice in KSFM. Infected cell monolayers were treated with 20 µg/ml penicillin G and 200 µg/ml gentamicin or without antibiotic for 45 minutes beginning 1 h 15 min post infection in order to determine the internalized and total associated GAS, respectively, at 2 h post infection. At 2 h post infection, intracellular and cell-associated bacteria were quantified as described previously [15]. Supernatant samples were also collected to determine the cell cytotoxicity due to GAS by measurement of lactate dehydrogenase (LDH) release (Roche). To determine intracellular survival, infected monolayers that had been treated with antibiotics were washed at 2 h post infection and replenished with fresh medium containing penicillin G (1 µg/ml). The infected monolayers were incubated for 4 h, 6 h, 8 h or 24 h post infection, at which times the total intracellular CFU were determined as above. For assays involving GAS strains harboring a plasmid, bacterial cultures were grown in the presence of antibiotic, and subsequent infections were performed in the absence of selection. Replica plating from the bacterial output plates onto antibiotic-containing medium confirmed plasmid maintenance during the assay. Intracellular survival assays using the inducible SLO (iSLO) complementation system were performed in the presence of 1 mM IPTG.

### Confocal microscopy

OKP7 cells were infected with GAS as described above. Antibiotics were omitted to avoid uptake and analysis of dead bacteria. For end points later than 2 h, cells were washed at 2 h post infection in PBS, and fresh KSFM was added for further incubation. At the end of each incubation, monolayers were washed with PBS and, when appropriate, extracellular GAS were stained with Alexa Fluor 660-conjugated anti-GAS IgG at 4°C for 15 min. Samples were then fixed and permeabilized by incubation in ice-cold methanol at −20°C for 5–10 minutes. Cells were then washed in PBS and incubated with the appropriate primary and secondary antibodies at room temperature in the dark for 1 h. Slides were mounted using Prolong Gold (Molecular Probes) and stored for 16–24 h at room temperature in the dark prior to imaging. Confocal microscopy was performed at the Harvard Digestive Diseases Center core facility as previously described [15]. Images were acquired and analyzed using Slidebook 5 (Intelligent Imaging Innovations, Denver, CO). For quantification, bacterial co-localization with endosomal markers was determined from three independent experiments, and at least 100 intracellular bacteria were counted for each experiment.

### Electron microscopy

OKP7 cells were infected for 3 h as described above (Confocal microscopy), immersed in fixative solution (0.1 M sodium cacodylate, 2% paraformaldehyde, 2.5% gluteraldehyde) for 30 min at room temperature, and then processed and imaged at the Electron Microscopy Core facility at Beth Israel Deaconess Medical Center (http://www.bidmc.org/Research/Core-Facilities/Electron-Microscopy-Core.aspx).

### Measurement of SLO and NADase activity

SLO activity was measured by determination of the hemolytic titers of culture supernatants from liquid cultures of GAS taken at early stationary phase, as described previously (Ruiz *et al*. 1998). NADase activities of bacterial culture supernatants and of the cytosolic fraction of eukaryotic cells exposed to GAS were determined as described previously [27].

### Statistical analysis

Statistical significance of differences between experimental conditions was determined by Student's t-test. P values of less than 0.05 were considered significant and are indicated by an asterisk (*) in figures.

## Supporting Information

Figure S1 **Intracellular survival of GAS in HeLa cells and cytotoxic effects of GAS on HeLa and OKP7 cells.** **A.** Intracellular survival in HeLa was determined as described above for strains 188 and 188SLO- (SLO-). Data represent mean±SD of three independent experiments. *, P<0.001. **B.** LDH release was measured at 2 h post-infection to determine cytotoxicity to HeLa cells due to GAS. **C.** LDH release from OKP7 cells infected with 188 and 188SLO- at 2 h, 6 h, 12 h and 24 h post-infection. Intracellular survival results were not considered reliable when LDH release exceeded 5% at 2 h post-infection (i.e., for HeLa cells infected with strain 188).
(PDF)

Figure S2 **Induction of autophagy in OKP7 cells by GAS or purified recombinant SLO.** **A.** GAS strain JRS4 and JRS4SLO- enter autophagosome-like compartments in OKP7 oropharyngeal keratinocytes. **B.** In HeLa cells, GAS localization to autophagosomes is SLO-dependent. HeLa cells stably expressing EGFP-LC3 were exposed to GAS strains 188 or 188SLO- (SLO-), and the association between EGFP-LC3 and GAS was assessed by confocal microscopy at 3 h post-infection as described above. Scale bar = 10 µm. **C.** Exposure of OKP7 cells to GAS strains that produce SLO (188, SLO-, NADase-, and SLS-) is associated with an increase in LC3-II. Left, lysates of uninfected cells or cells exposed to GAS strains for 2 h were probed with LC3 antibody. Right, cells were incubated with various amounts of recombinant SLO. An increase in the ratio of LC3-II∶LC3-I indicates increased autophagosome formation.
(PDF)

Figure S3 **siRNA knockdown of** ***becn1*** **(Beclin 1).** Lysates from OKP7 cells treated with non-targeting siRNA (non-target) or an siRNA pool directed against Beclin 1 (*becn1*) were analyzed by Western blot for Beclin 1 production. GAPDH was used as a loading control.
(PDF)

Figure S4 **A GAS strain engineered to secrete the endogenous NADase inhibitor IFS exhibits impaired intracellular survival.** **A.** Intracellular survival of strain 188 expressing a secreted FLAG-tagged GAS NADase inhibitor IFS from an expression vector (188(pSEC-IFS)) or strain 188 or 188NADase- carrying empty vector (188(pDL278) or NADase-(pDL278), respectively). *, P<0.001. Data represent the mean±SD of three independent experiments. **B.** Quantification of NADase activity in culture supernatants from GAS strains 188(pDL278), NADase-(pDL278), and 188(pSEC-IFS). **C.** Western blot for NADase and FLAG of culture supernatants described in (B). Expression and secretion of FLAG-tagged IFS (strain 188(pSEC-IFS)) eliminated extracellular NADase activity by GAS strain 188 despite the presence of wild type levels of NADase secretion by Western blot.
(PDF)

Table S1 **GAS strains used in this study.**
(PDF)

Table S2 **Oligonucleotide primers used in this study.**
(PDF)
