# Supplementary material for: Streptolysin O and its Co-Toxin NAD-glycohydrolase Protect Group A Streptococcus from Xenophagic Killing
Source: PLoS Pathog. 2013 Jun 6;9(6):e1003394. doi: 10.1371/journal.ppat.1003394 (PMC3675196; doi:10.1371/journal.ppat.1003394)
Supplement: Figure S1 — Intracellular survival of GAS in HeLa cells and cytotoxic effects of GAS on HeLa and OKP7 cells. A. Intracellular survival in HeLa was determined as described above for strains 188 and 188SLO- (SLO-). Data represent mean±SD of three independent experiments. *, P<0.001. B. LDH release was measured at 2 h post-infection to determine cytotoxicity to HeLa cells due to GAS. C. LDH release from OKP7 cells infected with 188 and 188SLO- at 2 h, 6 h, 12 h and 24 h post-infection. Intracellular survival results were not considered reliable when LDH release exceeded 5% at 2 h post-infection (i.e., for HeLa cells infected with strain 188). (PDF) [file ppat.1003394.s001.pdf]

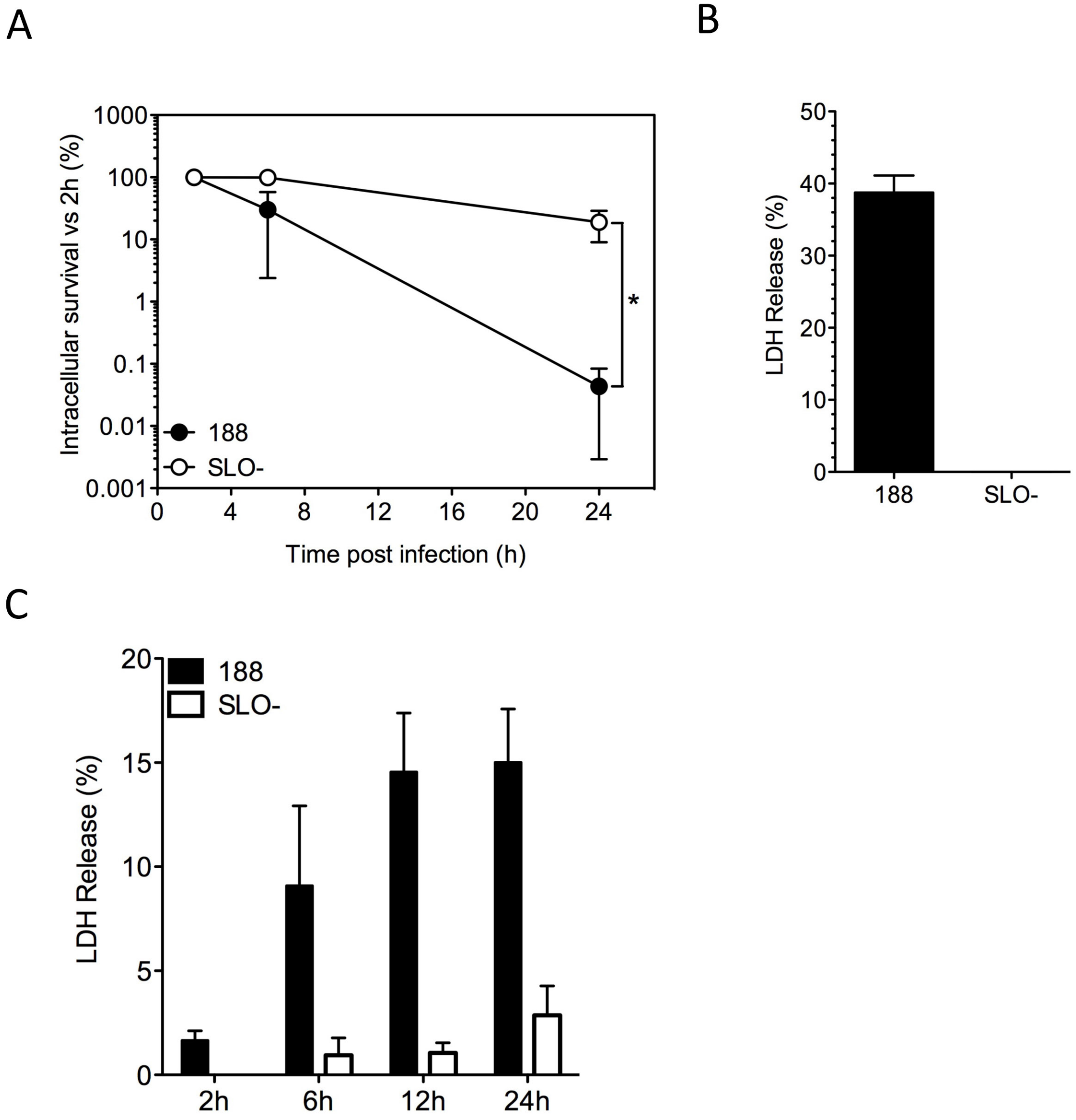

Figure S1. Intracellular survival of GAS in HeLa cells and cytotoxic effects of GAS on HeLa and OKP7 cells.

A. Intracellular survival in HeLa was determined as described above for strains 188 and 188SLO- (SLO-). Data represent mean±SD of three independent experiments. *, P<0.001. **B.** LDH release was measured at 2h post-infection to determine cytotoxicity to HeLa cells due to GAS. **C.** LDH release from OKP7 cells infected with 188 and 188SLO- at 2h, 6h, 12h and 24h post-infection. Intracellular survival results were not considered reliable when LDH release exceeded 5% at 2h post-infection (i.e., for HeLa cells infected with strain 188).
