# Supplementary material for: Streptolysin O and its Co-Toxin NAD-glycohydrolase Protect Group A Streptococcus from Xenophagic Killing
Source: PLoS Pathog. 2013 Jun 6;9(6):e1003394. doi: 10.1371/journal.ppat.1003394 (PMC3675196; doi:10.1371/journal.ppat.1003394)
Supplement: Figure S2 — Induction of autophagy in OKP7 cells by GAS or purified recombinant SLO. A. GAS strain JRS4 and JRS4SLO- enter autophagosome-like compartments in OKP7 oropharyngeal keratinocytes. B. In HeLa cells, GAS localization to autophagosomes is SLO-dependent. HeLa cells stably expressing EGFP-LC3 were exposed to GAS strains 188 or 188SLO- (SLO-), and the association between EGFP-LC3 and GAS was assessed by confocal microscopy at 3 h post-infection as described above. Scale bar = 10 µm. C. Exposure of OKP7 cells to GAS strains that produce SLO (188, SLO-, NADase-, and SLS-) is associated with an increase in LC3-II. Left, lysates of uninfected cells or cells exposed to GAS strains for 2 h were probed with LC3 antibody. Right, cells were incubated with various amounts of recombinant SLO. An increase in the ratio of LC3-II∶LC3-I indicates increased autophagosome formation. (PDF) [file ppat.1003394.s002.pdf]

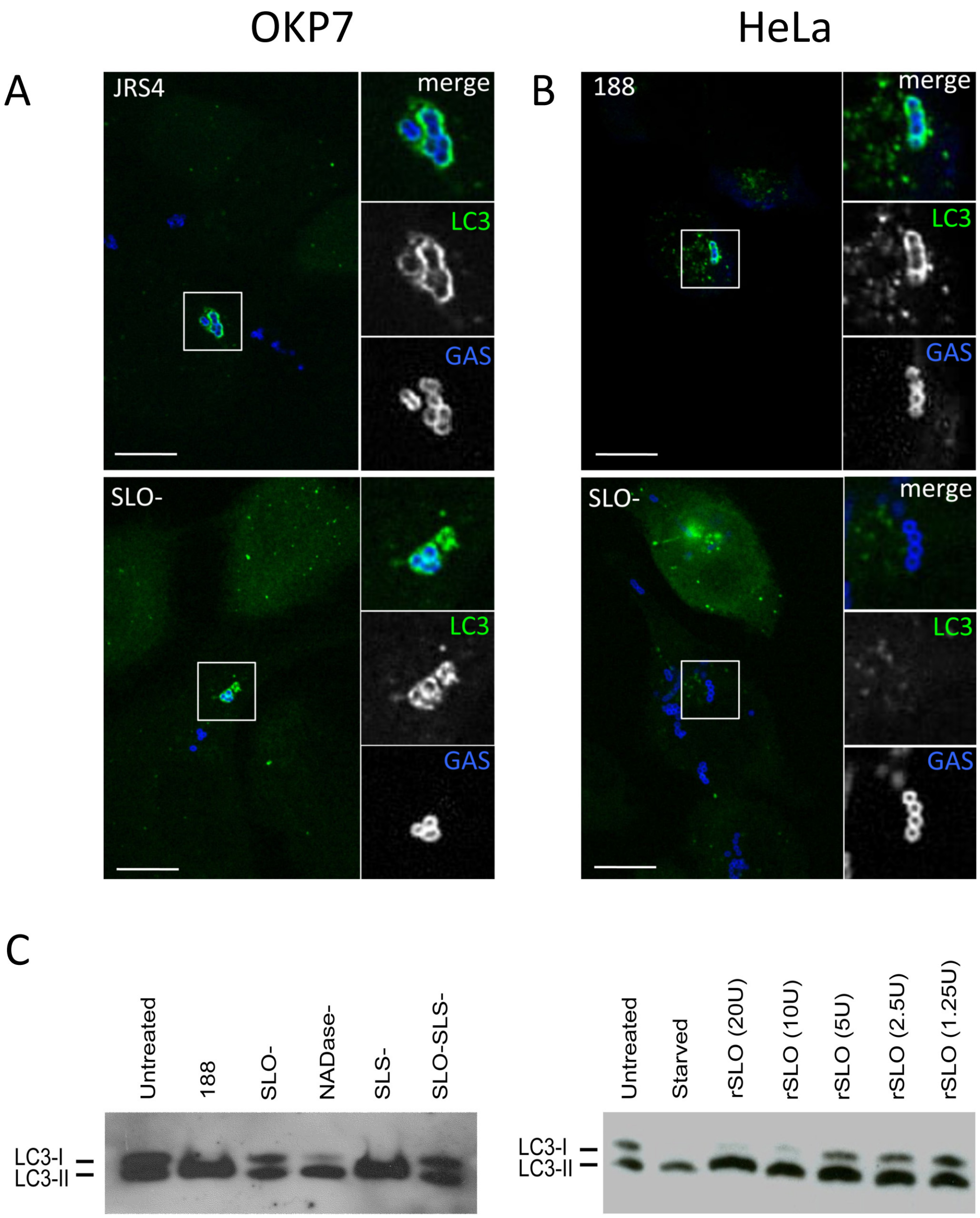

Figure S2. Induction of autophagy in OKP7 cells by GAS or purified recombinant SLO.

A. GAS strain JRS4 and JRS4SLO- enter autophagosome-like compartments in OKP7 oropharyngeal keratinocytes. **B.** In HeLa cells, GAS localization to autophagosomes is SLO-dependent. HeLa cells stably expressing EGFP-LC3 were exposed to GAS strains 188 or 188SLO- (SLO-), and the association between EGFP-LC3 and GAS was assessed by confocal microscopy at 3h post-infection as described above. Scale bar = 10 μm. **C.** Exposure of OKP7 cells to GAS strains that produce SLO (188, SLO-, NADase-, and SLS-) is associated with an increase in LC3-II. Left, lysates of uninfected cells or cells exposed to GAS strains for 2h were probed with LC3 antibody. Right, cells were incubated with various amounts of recombinant SLO. An increase in the ratio of LC3-II:LC3-I indicates increased autophagosome formation.
