# Supplementary material for: Streptolysin O and its Co-Toxin NAD-glycohydrolase Protect Group A Streptococcus from Xenophagic Killing
Source: PLoS Pathog. 2013 Jun 6;9(6):e1003394. doi: 10.1371/journal.ppat.1003394 (PMC3675196; doi:10.1371/journal.ppat.1003394)
Supplement: Figure S3 — siRNA knockdown of becn1 (Beclin 1). Lysates from OKP7 cells treated with non-targeting siRNA (non-target) or an siRNA pool directed against Beclin 1 (becn1) were analyzed by Western blot for Beclin 1 production. GAPDH was used as a loading control. (PDF) [file ppat.1003394.s003.pdf]

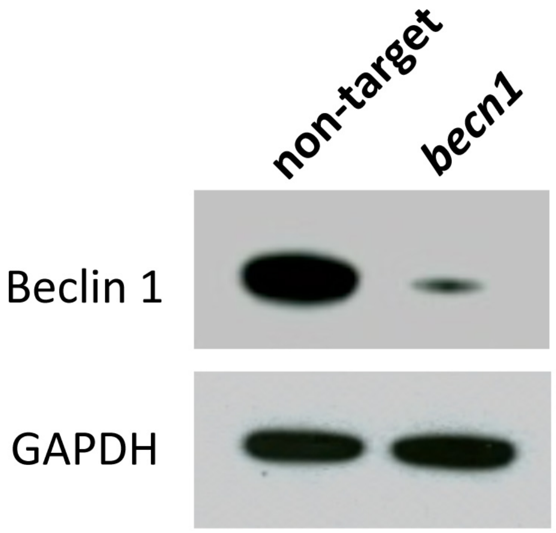

Figure S3. siRNA knockdown of *becn1* (Beclin 1).

Lysates from OKP7 cells treated with non-targeting siRNA (non-target) or an siRNA pool directed against Beclin 1 (*becn1*) were analyzed by Western blot for Beclin 1 production. GAPDH was used as a loading control.
