# Supplementary material for: Streptolysin O and its Co-Toxin NAD-glycohydrolase Protect Group A Streptococcus from Xenophagic Killing
Source: PLoS Pathog. 2013 Jun 6;9(6):e1003394. doi: 10.1371/journal.ppat.1003394 (PMC3675196; doi:10.1371/journal.ppat.1003394)
Supplement: Figure S4 — A GAS strain engineered to secrete the endogenous NADase inhibitor IFS exhibits impaired intracellular survival. A. Intracellular survival of strain 188 expressing a secreted FLAG-tagged GAS NADase inhibitor IFS from an expression vector (188(pSEC-IFS)) or strain 188 or 188NADase- carrying empty vector (188(pDL278) or NADase-(pDL278), respectively). *, P<0.001. Data represent the mean±SD of three independent experiments. B. Quantification of NADase activity in culture supernatants from GAS strains 188(pDL278), NADase-(pDL278), and 188(pSEC-IFS). C. Western blot for NADase and FLAG of culture supernatants described in (B). Expression and secretion of FLAG-tagged IFS (strain 188(pSEC-IFS)) eliminated extracellular NADase activity by GAS strain 188 despite the presence of wild type levels of NADase secretion by Western blot. (PDF) [file ppat.1003394.s004.pdf]

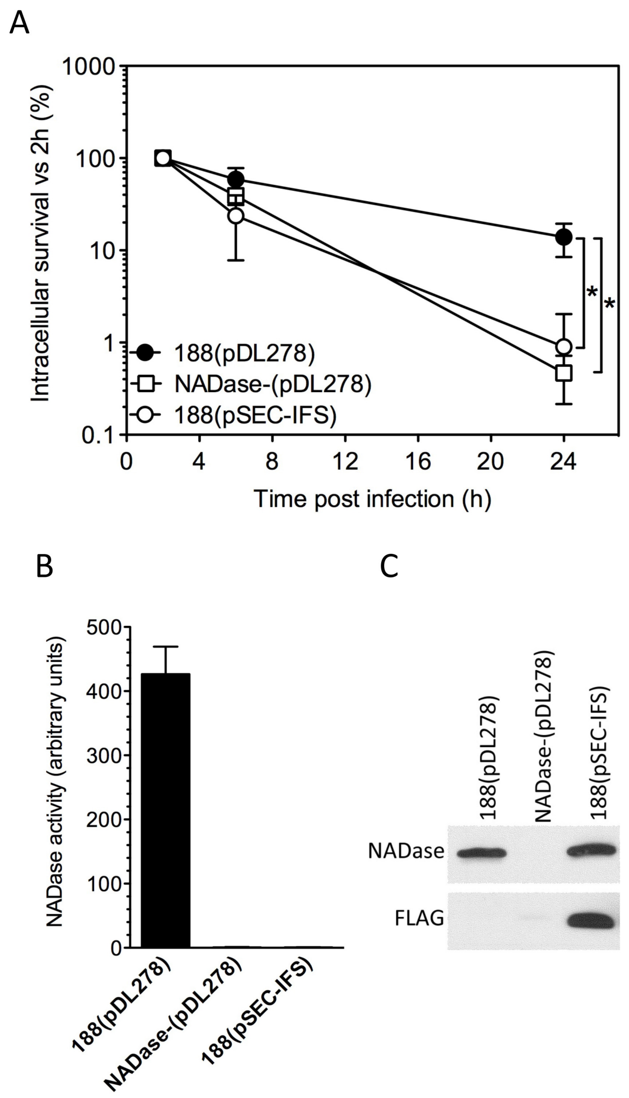

Figure S4. A GAS strain engineered to secrete the endogenous NADase inhibitor IFS exhibits impaired intracellular survival.

A. Intracellular survival of strain 188 expressing a secreted FLAG-tagged GAS NADase inhibitor IFS from an expression vector (188(pSEC-IFS)) or strain 188 or 188NADase- carrying empty vector (188(pDL278) or NADase-(pDL278), respectively). *, $P<0.001$. Data represent the mean±SD of three independent experiments. **B.** Quantification of NADase activity in culture supernatants from GAS strains 188(pDL278), NADase-(pDL278), and 188(pSEC-IFS). **C.** Western blot for NADase and FLAG of culture supernatants described in (B). Expression and secretion of FLAG-tagged IFS (strain 188(pSEC-IFS)) eliminated extracellular NADase activity by GAS strain 188 despite the presence of wild type levels of NADase secretion by Western blot.
