# Supplementary material for: Streptolysin O and its Co-Toxin NAD-glycohydrolase Protect Group A Streptococcus from Xenophagic Killing
Source: PLoS Pathog. 2013 Jun 6;9(6):e1003394. doi: 10.1371/journal.ppat.1003394 (PMC3675196; doi:10.1371/journal.ppat.1003394)
Supplement: Table S1 — GAS strains used in this study. (PDF) [file ppat.1003394.s005.pdf]

Table S1. GAS strains used in this study.

Strain	Characteristics	Source/reference
950771 (771)	Encapsulated necrotizing fasciitis isolate	[33]
771SLO-	*slo*-negative deletion mutant of 771	[27]
771NADase-	*nga*-negative deletion mutant of 771	[27]
771SLO(Y255A)	Y255A point mutant of *slo*, deficient in pore formation but competent for NADase translocation by SLO	This study
188	Unencapsulated mutant of 950771	[33]
188SLO-	*slo*-negative deletion mutant of 188	[47]
188NADase-	*nga*-negative deletion mutant of 188	[14]
188SLS-	*sagA*-negative deletion mutant of 188	This study
188SLO-SLS-	*slo and sagA*-negative deletion mutant of 188	This study
188NADase(G330D)	G330D point mutant of *nga*, lacking NAD-glycohydrolase activity, in strain 188	This study
188SLO(Y255A)	Y255A point mutant of *slo*, deficient in pore formation but competent for NADase translocation by SLO, in strain 188	This study
188(pSIV)	188 containing streptococcal IPTG-inducible vector (pSIV). Erm^R	[15]
188SLO-(pSIV)	188SLO- containing pSIV. Erm^R	[15]
188SLO-(piSLO)	188SLO- containing IPTG-inducible SLO in pSIV. Erm^R	[15]
188(pDL278)	188 containing empty pDL278 vector. Spc^R	This study
188NADase-(pDL278)	188NADase- containing empty pDL278 vector. Spc^R	This study
188(pSEC-IFS)	188 containing secreted IFS (SEC-IFS) under constitutive control of P*guaB* in pDL278. Spc^R	This study
JRS4	Derivative of the M6 strain D471 from the Rockefeller University collection	[44]
JRS4SLO-	*slo*-negative deletion mutant of JRS4 (SLO1)	[43]
JRS4NADase-	*nga*-negative deletion mutant of JRS4 (SPN1)	[28]

Abbreviations: Erm, erythromycin; Spc, spectinomycin.
* Reference numbers refer to reference list of the article.
