# Supplementary material for: Streptolysin O and its Co-Toxin NAD-glycohydrolase Protect Group A Streptococcus from Xenophagic Killing
Source: PLoS Pathog. 2013 Jun 6;9(6):e1003394. doi: 10.1371/journal.ppat.1003394 (PMC3675196; doi:10.1371/journal.ppat.1003394)
Supplement: Table S2 — Oligonucleotide primers used in this study. (PDF) [file ppat.1003394.s006.pdf]

Table S2. Oligonucleotide primers used in this study. Restriction sites are underlined.

Primer	Sequence (5′-3′)
sloY255AF	CTGCCAGAACACAAGCTACTGAATCAATGGTATATTC
sloY255AR	GAATATACCATTGATTCAGTAGCTTGTGTTCTGGCAG
ngaG330DF	AAGACGTCGATAGCGGAAAATATAGCGAT
ngaG330DR	TATAAAAGACGTCGATAGCGGAAAATATAGC
slo_up	CCGGGATCCGAAAATGCAAGATAGAATGCAAGATAGGA
slo_down	GTATGCTGCAATCATCACCTTCTTTTCACCGTCGACCGG
nga_up	CCGGGATCCCAGAGGTCACTTTTGGCAATATGG
nga_down	CCGGTCGACCTAAAATGTTTCTATTGTTC
SigF	CCGGCTAGCAATAAAAAAACATTTAAAAAATAC
SigR	CGGGATCCAGCATTAGCAGTAACAAGGTTACCAATGAT
ifsF	CGGGATCCGATTATAAAGATGATGACGATAAAT
ifsR	CCGGTCGACCTAAAATGTTTTTATTGTTCTTTCGACCATATC
